# Structure of the MICU1–MICU2 heterodimer provides insights into the gatekeeping threshold shift

**DOI:** 10.1107/S2052252520001840

**Published:** 2020-02-27

**Authors:** Jongseo Park, Youngjin Lee, Taein Park, Jung Youn Kang, Sang A Mun, Minwoo Jin, Jihyeong Yang, Soo Hyun Eom

**Affiliations:** a School of Life Sciences, Gwangju Institute of Science and Technology (GIST), Buk-gu, Gwangju 61005, Republic of Korea; b Steitz Center for Structural Biology, Gwangju Institute of Science and Technology (GIST), Buk-gu, Gwangju 61005, Republic of Korea; c Infection and Immunity Research Laboratory, Metabolic Regulation Research Center, Korea Research Institute of Bioscience and Biotechnology (KRIBB), Daejeon 34141, Republic of Korea; d Department of Chemistry, Gwangju Institute of Science and Technology (GIST), Buk-gu, Gwangju 61005, Republic of Korea

**Keywords:** mitochondrial calcium uptake, MICU1–MICU2 heterodimer, MCU gatekeepers, cooperativity, Ca^2+^ sensing proteins, X-ray crystallography

## Abstract

Mitochondrial calcium uptake proteins 1 and 2 (MICU1 and MICU2) mediate mitochondrial Ca^2+^ influx via the mitochondrial calcium uniporter (MCU). Its molecular action for Ca^2+^ uptake is tightly controlled by the MICU1–MICU2 heterodimer, which comprises Ca^2+^ sensing proteins which act as gatekeepers at low [Ca^2+^] or facilitators at high [Ca^2+^]. However, the mechanism underlying the regulation of the Ca^2+^ gatekeeping threshold for mitochondrial Ca^2+^ uptake through the MCU by the MICU1–MICU2 heterodimer remains unclear. In this study, we determined the crystal structure of the apo form of the human MICU1–MICU2 heterodimer that functions as the MCU gatekeeper. MICU1 and MICU2 assemble in the face-to-face heterodimer with salt bridges and me­thio­nine knobs stabilizing the heterodimer in an apo state. Structural analysis suggests how the heterodimer sets a higher Ca^2+^ threshold than the MICU1 homodimer. The structure of the heterodimer in the apo state provides a framework for understanding the gatekeeping role of the MICU1–MICU2 heterodimer.

## Abstract 2

Structural analysis of the apo form of the human MICU1–MICU2 heterodimer suggests how the heterodimer sets a higher Ca^2+^ threshold than the MICU1 homodimer. This provides a framework for understanding the gatekeeping role of the MICU1–MICU2 heterodimer.

## 1. Introduction   

Mitochondrial calcium homeostasis plays an essential role in modulating cellular functions including energy synthesis, signal transduction, and mitochondrial fusion and fission (Viola & Hool, 2010 ▸; Giacomello *et al.*, 2007 ▸; Rizzuto *et al.*, 2012 ▸). Its malfunction promotes cell death and triggers several diseases such as cerebrovascular or cardiovascular disease, motor disorders and cancer development. In particular, mitochondrial accumulation of large quantities of Ca^2+^ triggers excessive reactive oxygen species production, and eventually causes apoptosis and necrosis in pathological processes (Rizzuto *et al.*, 2012 ▸; Kinnally *et al.*, 2011 ▸; Tamargo & López-Sendón, 2011 ▸; Ansley & Wang, 2013 ▸; Wang *et al.*, 2015 ▸; Clapham, 1995 ▸; Kamer & Mootha, 2015 ▸).

Thus, mitochondrial calcium homeostasis is tightly controlled by mitochondrial Ca^2+^ channels such as the Na^+^–Ca^2+^ exchanger, voltage-dependent anion channels (VDACs), the permeability transition pore (PTP) and the mitochondrial calcium uniporter (MCU) complex. The eukaryotic MCU complex comprises the MCU pore-forming subunit, an MCU paralog (MCUb), an essential MCU regulator (EMRE), MCU regulator 1 (MCUR1) localized in the inner mitochondrial membrane and mitochondrial calcium uptake (MICU) proteins in the inter-membrane space (Kirichok *et al.*, 2004 ▸; Sancak *et al.*, 2013 ▸; Tsai *et al.*, 2016 ▸; Mallilankaraman *et al.*, 2012*a* ▸; Perocchi *et al.*, 2010 ▸; Plovanich *et al.*, 2013 ▸; Vecellio Reane *et al.*, 2016 ▸; Tomar *et al.*, 2016 ▸).

MICU proteins have Ca^2+^ binding EF-hand motifs, which form homo- or hetero-oligomers, and function as gatekeepers in the Ca^2+^-free (apo) state and facilitators in the Ca^2+^-bound state (Mallilankaraman *et al.*, 2012*b* ▸; Csordás *et al.*, 2013 ▸; Hoffman *et al.*, 2013 ▸; Waldeck-Weiermair *et al.*, 2015 ▸; Liu *et al.*, 2016 ▸; Matesanz-Isabel *et al.*, 2016 ▸). MICU proteins have evolved from slime molds to humans and have been differentiated into MICU paralogs as well as an alternative splicing variant. Recent reports suggest that *Dictyostelium discoideum* has one MICU protein (*Dd*MICU), whereas *Arabidopsis thaliana* has two MICU proteins (*At*MICU1 and *At*MICU2) (Wagner *et al.*, 2015 ▸; Kovács-Bogdán *et al.*, 2014 ▸; Bick *et al.*, 2012 ▸). Intriguingly, humans have three MICU paralogs (MICU1, MICU2 and MICU3), as well as an alternative splicing variant (MICU1.1) depending on the tissues, and human MICU1 and MICU2 generally assemble as a heterodimer and regulate Ca^2+^ uptake of the MCU complex in most tissues (Perocchi *et al.*, 2010 ▸; Plovanich *et al.*, 2013 ▸; Vecellio Reane *et al.*, 2016 ▸; Paillard *et al.*, 2017 ▸).

Blocking or activation of mitochondrial Ca^2+^ (Ca^2+^_m_) uptake by the MCU complex is affected by Ca^2+^ binding to the MICUs participating in the MCU complex because a strong correlation between Ca^2+^ binding affinity and the MCU Ca^2+^ gatekeeping threshold for Ca^2+^_m_ uptake has been demonstrated (Kamer *et al.*, 2017 ▸). Indeed, the Ca^2+^ threshold for Ca^2+^_m_ uptake of the MCU, as set by the MICU1–MICU2 heterodimer, is 700 n*M*, which is similar to the Ca^2+^ binding affinity with *K*_d_ = 620 n*M*. Whereas the MICU1 homodimer sets the Ca^2+^ threshold for Ca^2+^_m_ uptake through the MCU at 350 n*M*, which is similar to the Ca^2+^ binding affinity with *K*_d_ ≃ 300 n*M* (Kamer *et al.*, 2017 ▸).

Loss of MICU1 and MICU2 functions diminishes the cross talk between the endoplasmic reticulum and mitochondria, and induces excessive Ca^2+^ uptake through the MCU (Payne *et al.*, 2017 ▸). This excessive Ca^2+^ uptake induces several diseases such as proximal myopathy, learning difficulties and progressive extrapyramidal movement disorders (Logan *et al.*, 2014 ▸). However, the structure of the heterodimers is not known and the mechanism underlying the regulation of Ca^2+^ uptake by the heterodimer through the MCU is also unclear, although the structures of the homodimers in MICU1, MICU2 and MICU3 have been reported previously (Wang *et al.*, 2014 ▸; Kamer *et al.*, 2019 ▸; Xing *et al.*, 2019 ▸; Wu *et al.*, 2019 ▸).

In this report, we present the crystal structure of an apo human MICU1–MICU2 heterodimer. We found a unique salt bridge, Asp231(MICU1)–Arg352(MICU2), within the heterodimer. Based on the structural and biochemical analysis, we can explain how the heterodimer shows a Ca^2+^ binding affinity lower than that of MICU1. Moreover, the structure of the MICU1–MICU2 heterodimer in the apo state provides insight into how the MICU1–MICU2 heterodimer has a higher Ca^2+^ threshold for Ca^2+^_m_ uptake through the MCU rather than the MICU1 homodimer.

## 2. Results   

### 2.1. Overall structure of the MICU1–MICU2 heterodimer   

**Figure 1:**
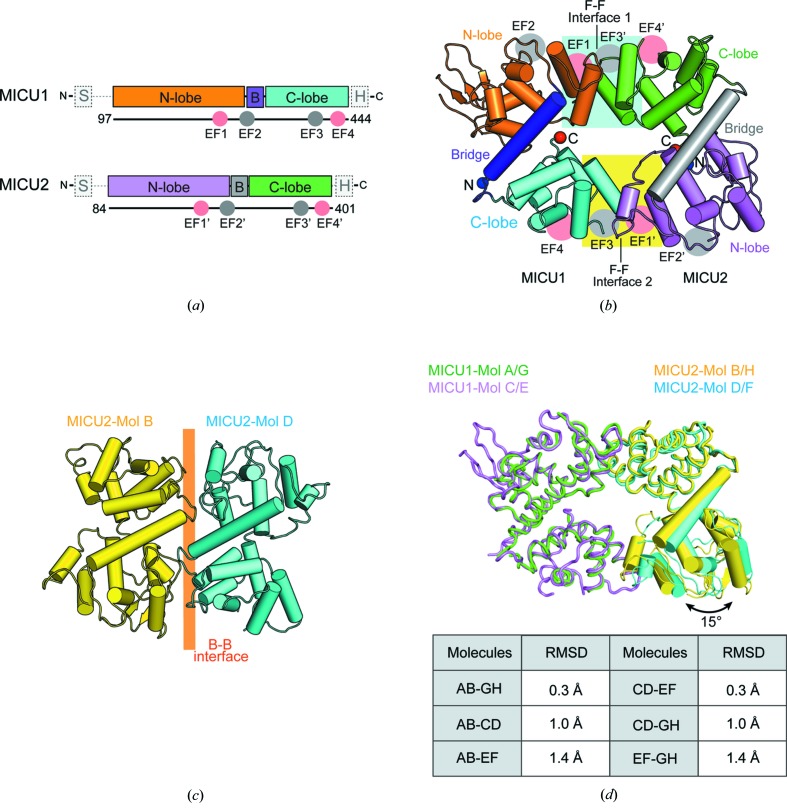
Overall structure of the MICU1–MICU2 heterodimer and the B–B dimer of MICU2 in an ASU. (*a*) Schematic diagram of MICU1 and MICU2. MICU1 and MICU2 consist of mitochondrial targeting sequences (S), N lobes, bridge helices (B), C lobes, and C-terminal helices (H). MICU1 and MICU2 contain two canonical EF hands (EF-hand 1 and EF-hand 4) and two non-canonical EF hands (EF-hand 2 and EF-hand 3). (*b*) A cartoon representation of the overall structure of the MICU1–MICU2 heterodimer. Interface 1, comprising the MICU1 N lobe and the MICU2 C lobe, is highlighted in the cyan square. Interface 2, comprising the MICU1 C lobe and the MICU2 N lobe, is highlighted in the yellow square. (*c*) A cartoon representation of the B–B MICU2 dimer in the ASU. (*d*) A ribbon and cylindrical diagram of the superimposed AB and CD dimers based on MICU1, and a table of RMSD values between each heterodimer. MICU1 is colored green or violet and MICU2 colored yellow or cyan. The tilt angle is marked with a black arrow.

We crystallized the heterodimer including wild-type MICU1 and a selenome­thio­nine (SeMet) derivative of MICU2, and solved the 3.1 Å resolution crystal structure of the MICU1–MICU2 heterodimer using single-wavelength anomalous dispersion (Table 1 ▸). Four MICU1–MICU2 heterodimers were observed per asymmetric unit (ASU) (see Fig. S1 in the Supporting information). The heterodimers were formed by a face-to-face (F–F) interface similar to other MICU homodimer structures [Figs. 1 ▸(*a*) and 1 ▸(*b*)] (Wang *et al.*, 2014 ▸; Kamer *et al.*, 2019 ▸; Xing *et al.*, 2019 ▸; Wu *et al.*, 2019 ▸). Unlike MICU homodimer structures, the MICU1–MICU2 heterodimer comprised two interfaces, interfaces 1 and 2, as a result of the asymmetry of the heterodimer. Interestingly, a back-to-back (B–B) interaction was observed among heterodimers through MICU2 in an ASU, which was also observed between MICU2 homodimers [Fig. 1 ▸(*c*)] (Xing *et al.*, 2019 ▸). When we superimposed four MICU1–MICU2 heterodimers in an ASU, the topology of the four heterodimers was slightly different. We classified the heterodimers into two classes, an AB dimer and a CD dimer, based on the root-mean-square deviation (RMSD) [Fig. 1 ▸(*d*)]. When we compared the AB and CD dimers, interface 1 was well aligned (RMSD = 0.5 Å), whereas interface 2 was more variable and the N lobe of MICU2 was tilted by 15° [Fig. 1 ▸(*d*)]. Therefore, the interfaces of the MICU1–MICU2 heterodimer contained a rigid interface 1 and a variable interface 2.

### 2.2. Structural comparison with MICU homodimers   

**Figure 2:**
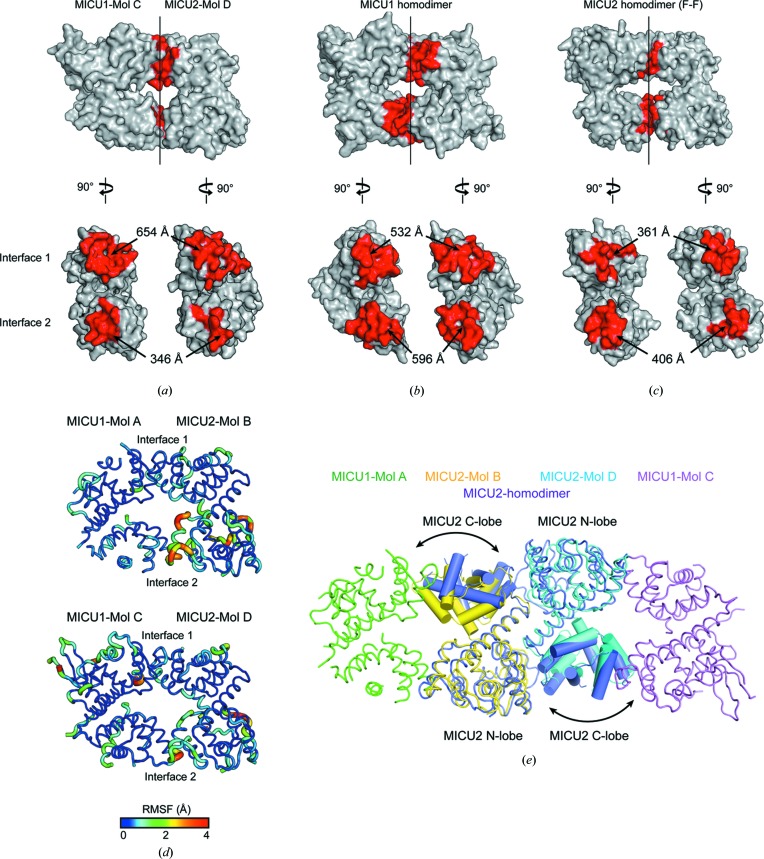
Structural comparison between the MICU1–MICU2 heterodimer and other homodimers of MICU1 and MICU2. (*a*)–(*c*) Space-filling model of the MICU1–MICU2 heterodimer (Mol CD), an apo MICU1 homodimer (PDB ID 4nsc; Wang *et al.*, 2014 ▸) and an apo MICU2 homodimer (F–F) (PDB ID 6agh; Xing *et al.*, 2019 ▸). The red regions indicate the interface areas. (*d*) Representations of the RMSF based on the ensemble refinements of MICU1 (Mol A and C) or MICU2 (Mol B and D) in the MICU1–MICU2 heterodimer. (*e*) A ribbon and cylindrical diagram of superimposed apo MICU2 (purple) (PDB ID 6agh) and B–B dimer (yellow and cyan) among MICU1–MICU2 heterodimers in the ASU. The tilted regions are marked with black arrows.

The MICU1–MICU2 heterodimer interface was asymmetric unlike other MICU1 or MICU2 homodimers and the topologies of the heterodimer were variable in the ASU. To understand the structural differences between the MICU1–MICU2 heterodimer and MICU1 or MICU2 homodimers, we compared the interfaces of the MICU1–MICU2 heterodimer and the homodimers of MICU1 (F–F) or MICU2 (F–F and B–B). Compared with MICU1 (F–F) and MICU2 (F–F) homodimers, the MICU1–MICU2 heterodimer (CD dimer) had a wider interface area in interface 1 but a narrower interface area in interface 2 [Figs. 2 ▸(*a*)–2(*c*)]. In addition, we calculated the binding energy for the two interfaces using *PRODIGY* (Xue *et al.*, 2016 ▸). The predicted binding energies for interface 1 and interface 2 are −7.6 and −5.2 kcal mol^−1^, showing that interface 1 is more energetically stable (Supplementary Fig. S2). Root-mean-square fluctuation (RMSF) analysis showed that interface 1 was more rigid than interface 2 [Fig. 2 ▸(*d*)].

In the case of the B–B dimer comprising MICU2 (Mol BD) among the MICU1–MICU2 heterodimers in the ASU, the overall topology was similar to the B–B dimer of the apo state MICU2 homodimer [Fig. 2 ▸(*e*)] (Xing *et al.*, 2019 ▸). However, the interface area in the B–B dimer of Mol BD was 1.5 times smaller than that of the B–B dimer among MICU2 homodimers (Fig. S2). The RMSD value of two MICU2 subunits in a heterodimer and a homodimer was 1.5 Å, whereas the individual N lobe or C lobe of each MICU2 was well aligned with the RMSD values of 0.5 Å or 0.8 Å, respectively. This deviation originated in the variable topology between the N lobe and C lobe of MICU2 in the heterodimer and the homodimer [Fig. 2 ▸(*e*)]. Thus, MICU2 in the heterodimer had a different topology between the N lobe and C lobe compared with MICU2 in the homodimer because of interaction with MICU1 in the MICU1–MICU2 heterodimer.

### 2.3. Structural details of the MICU1–MICU2 heterodimer interfaces   

**Figure 3:**
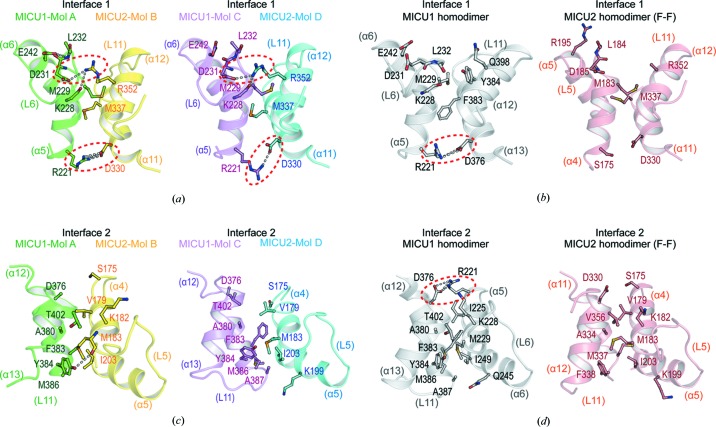
Interaction interfaces of the MICU1–MICU2 heterodimer and comparison with MICU homodimers. Cartoon representations of interfaces 1 and 2 in the MICU1–MICU2 heterodimer (Mol AB and CD) and homodimers in MICU1 or MICU2. (*a*), (*b*) The side chains of residues participating in interface 1 interactions of (*a*) Mol AB, CD, and (*b*) the homodimers in MICU1 and MICU2 are shown in the stick form. (*c*), (*d*) The side chains of residues participating in interface 2 interactions of (*c*) Mol AB, CD, and (*d*) homodimers in MICU1 and MICU2 are shown in the stick form. The white dashed lines and red dotted circles denote electrostatic interactions and salt bridges, respectively.

While homodimers in MICU1 and MICU2 had a symmetrical dimer interface, the MICU1–MICU2 heterodimer had an asymmetric dimer interface with interfaces 1 and 2. To understand the significance of the asymmetry of the dimer interface in the MICU1–MICU2 heterodimer, we analyzed the dimer interface in detail. Compared with the homodimers in MICU1 and MICU2, only structural differences in the interloop (L6) of MICU1 EF-hand 1 were observed in interface 1 comprising MICU1 EF-hand 1 and MICU2 EF-hand 3 (Fig. S3). The primary interactions between MICU1 and MICU2 were two salt bridges involving the highly conserved side chains of Arg221(MICU1)–Asp330(MICU2) and Asp231(MICU1)–Arg352(MICU2) [Figs. 3 ▸(*a*), S4 and S5]. These two salt bridges were present in both the AB and CD dimers. The Arg221(MICU1)–Asp330(MICU2) salt bridge was located at the same position as the Arg221–Asp376 salt bridge in the apo state of the human MICU1 homodimer [Fig. 3 ▸(*b*)] (Wang *et al.*, 2014 ▸). Indeed, the Arg221–Asp376 salt bridge in the interface of the MICU1 homodimer is essential for dimerization in the apo state of the homodimer. The mutant (R221A–D376A), which prevents the formation of the salt bridge, did not form a homodimer. Furthermore, this mutant lost the MCU facilitator activity of MICU1 (Wang *et al.*, 2014 ▸). It appears that the MICU1 homodimer interface including Arg221–Asp376 is biologically relevant. Thus, the MICU1–MICU2 heterodimer interface, which has a similar interface to the MICU1 homodimer, is also biologically relevant. In contrast, the other salt bridge, Asp231(MICU1)–Arg352(MICU2), has never been observed in other MICU homodimer structures. Notably, the Asp231(MICU1) was located on the interloop (L6) of EF-hand 1 participating in the Ca^2+^ coordination, indicating that Ca^2+^ binding induces structural changes directly on interface 1. Arg352(MICU2) was identified as one of two important residues, Glu242(MICU1) and Arg352(MICU2), whose alanine mutation disturbs the heterodimerization of MICU1 and MICU2 in the apo state (Wu *et al.*, 2019 ▸). However, Glu242(MICU1) did not directly participate in interface 1 of the structure but stabilized the conformation of the interloop (L6) by forming a hydrogen bond through the backbone amide nitro­gen of Leu232 [Fig. 3 ▸(*a*)].

In addition to the salt bridges, interface 1 included several hydro­phobic interactions involving the highly conserved Met229(MICU1) and Met337(MICU2) [Figs. 3 ▸(*a*), S4 and S5]. Notably, two me­thio­nine residues protruded from their EF-hand like knob and fit into the hydro­phobic hole formed between the opposite EF-hand helices, consistent with the highest positive solvation energy calculated using the PDBePISA web server (http://www.ebi.ac.uk/pdbe/prot_int/) (Fig. S6). In the case of the MICU1 homodimer, Met229 was not able to protrude in the opposite direction because of Tyr384, which acts as a barrier [Fig. 3 ▸(*b*)]. Unlike the MICU1 homodimer, Met337 in the MICU2 homodimer participated in hydro­phobic interactions with the opposite EF-hand helices, although no ionic interactions were involved in the interface of the MICU2 homodimer [Fig. 3 ▸(*b*)]. Consistent with the analysis of interface 1, its interface area in the MICU1–MICU2 heterodimer (654 Å) was wider than that of the homodimers in MICU1 (532 Å) and MICU2 (361 Å). Thus, we suggested that the interaction of interface 1 in the MICU1–MICU2 heterodimer was stronger than that in the homodimers of MICU1 and MICU2 because of the additional salt bridge and two meth­ionine knobs. Consistently, interface 1 in the MICU1–MICU2 heterodimer had a wider interface area and stronger predicted binding energy than that in the homodimers of MICU1 and MICU2 (Fig. S2).

Unlike interface 1, interface 2 was only composed of several hydro­phobic residues including Phe383, Tyr384, Met386 and Ala387 in MICU1, and Val179, Met183, Lys199 and Ile203 in MICU2 [Figs. 3 ▸(*c*), S4 and S5]. Interface 2 did not include residues involved in ionic interaction similar to Arg221–Asp376 in the MICU1 homodimer [Figs. 3 ▸(*c*) and 3 ▸(*d*)]. In the case of interface 2, compared with homodimers of MICU1 and MICU2, structural differences were only observed in MICU2 EF-hand 1 in the MICU1–MICU2 heterodimer (Fig. S3). Consistent with the analysis of interface 2, the interface area of interface 2 in the MICU1–MICU2 heterodimer (346 Å) was narrower than that in the homodimers of MICU1 (596 Å) and MICU2 (406 Å). Thus, we suggested that the interaction of interface 2 in the MICU1–MICU2 heterodimer was weaker than that in the homodimers of MICU1 and MICU2. Consistently, interface 2 in the MICU1–MICU2 heterodimer had a narrower interface area and weaker predicted binding energy than that in the homodimers of MICU1 and MICU2 (Fig. S2).

Upon analysis of the interface area and predicted binding energy, interface 1 of the MICU1–MICU2 heterodimer had a wider interface area and stronger binding energy than that in the homodimers of MICU1 and MICU2. However, in interface 2, the MICU1–MICU2 heterodimer had a narrower interface area and weaker binding energy than that in the homodimers of MICU1 and MICU2. In addition, previous biochemical studies have indicated that the MICU1–MICU2 heterodimer is more stable (*K*_d_ = 224 n*M* in the apo state) compared with the homodimers of MICU1 and MICU2 (Wu *et al.*, 2019 ▸; Kamer *et al.*, 2017 ▸), implying that a strong interaction through interface 1 is important for MICU1–MICU2 heterodimerization. Thus, the MICU1–MICU2 heterodimer was found to be more stable than the homodimers, and the asymmetrical interface of the MICU1–MICU2 heterodimer showed the unique feature of a more stable interface 1 and a more flexible interface 2 compared with the homodimer interfaces.

### 2.4. Structural comparison with Ca^2+^-bound homodimer structure of MICU1   

The MICU1–MICU2 heterodimer set a Ca^2+^ threshold at 700 n*M* for Ca^2+^_m_ uptake through the MCU, which is at a concentration two times higher than that of the MICU1 homodimer (∼300 n*M*), which is consistent with the Ca^2+^ binding affinities of the MICU1–MICU2 heterodimer (*K*_d_ = 620 n*M*) (Kamer *et al.*, 2017 ▸). This affinity and threshold shift originated in the cooperative Ca^2+^ binding to the MICU1–MICU2 heterodimer (Kamer *et al.*, 2017 ▸). Accordingly, we investigated the cooperativity of the MICU1–MICU2 heterodimer and how the Ca^2+^ binding affinities of the heterodimer change.

**Figure 4:**
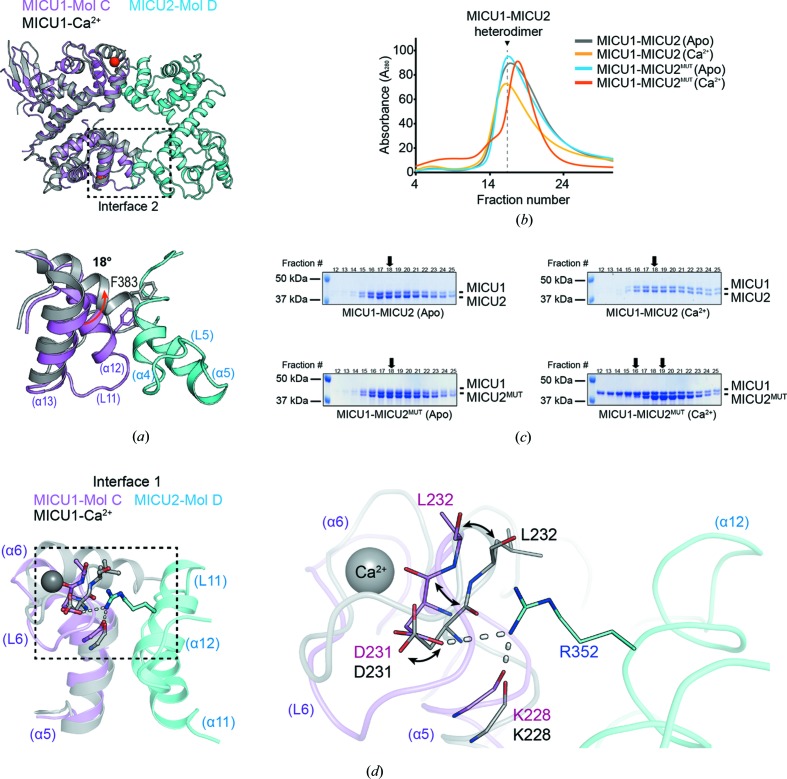
Structural and biochemical analysis for comparison with Ca^2+^-bound MICU1. (*a*) Cartoon representations of the superimposed apo heterodimer and one molecule of Ca^2+^-bound MICU1 (gray) (PDB ID 4nsd; Wang *et al.*, 2014 ▸), and a detailed view of interface 2 of the superimposed structure. The 18° tilt of α12 helix of MICU1 EF-hand 3 is indicated by a red arrow. The heterodimer is colored violet (MICU1) and cyan (MICU2). (*b*) SEC profile of the MICU1–MICU2 or MICU1–MICU2^MUT^ heterodimer, and (*c*) its SDS–PAGE results in the absence (marked by Apo) or presence of Ca^2+^ (marked by Ca^2+^). The black arrows indicate the peak fractions of each SDS gel. The two black arrows in the MICU1–MICU2^MUT^(Ca^2+^) heterodimer indicate the peak fractions of MICU1 and MICU2 (left and right), respectively. (*d*) A cartoon representation of the superimposed interface 1 of the apo MICU1–MICU2 heterodimer with one molecule of Ca^2+^-bound MICU1 (gray) (PDB ID 4nsd) based on MICU1 in the heterodimer. The conformational changes of MICU1 EF-hand 1 including the Asp231 and Leu232 residue are indicated by a stick representation and black arrows.

Ca^2+^ binding to the MICU1–MICU2 heterodimer can occur either in an independent or a cooperative manner. If Ca^2+^ independently binds to MICU1 and MICU2 in the heterodimer, the intermediate state in the heterodimer might exist and that might be the MICU1(Ca^2+^ bound)–MICU2(apo) state because of the high Ca^2+^ affinity for MICU1. However, this hypothetical intermediate state is not possible because of clashes [Fig. 4 ▸(*a*)]. Thus, the intermediate states of the MICU1–MICU2 heterodimer are not possible, and a Ca^2+^-bound MICU1–MICU2 heterodimer is formed when Ca^2+^ binds to MICU1 and MICU2 cooperatively.

In addition to the structural analysis, we performed a biochemical study to verify whether the intermediate state can possibly exist. We designed a MICU2 mutant (MICU2^MUT^) whose two calcium binding sites are mutated (D185A/E196K for EF-hand 1, D375A/E386K for EF-hand 4). First, we prepared mixed MICU1 and MICU2^MUT^ in the apo state, and compared them with the wild-type MICU1–MICU2 heterodimer in terms of size [Figs. 4 ▸(*b*) and 4 ▸(*c*)]. The MICU1 and MICU2^MUT^ in the apo state behaved similarly to the wild-type MICU1–MICU2 heterodimer in size-exclusion chromatography (SEC), and the ratio of MICU1 and MICU2 intensities in the profile were equal. Thus, we concluded that MICU1 and MICU2^MUT^ in the apo state formed a stable heterodimer. To mimic the MICU1(Ca^2+^ bound)–MICU2(apo) heterodimer state, we added Ca^2+^ to the mixture of MICU1 and MICU2^MUT^ to a final concentration of 5 m*M*. Unlike the apo state, the mixture of MICU1(Ca^2+^ bound) and MICU2^MUT^(apo) was eluted differently in SEC compared with the apo state of the MICU1 and MICU2^MUT^ [Fig. 4 ▸(*b*)], and the ratio of MICU1(Ca^2+^ bound) and MICU2^MUT^(apo) intensities in the profile were not equal [Fig. 4 ▸(*c*)], implying that the mixture of MICU1(Ca^2+^ bound) and MICU2^MUT^(apo) did not form a stable heterodimer. Thus, intermediate states of the MICU1(Ca^2+^ bound) and MICU2(apo) heterodimer are not stable. Consequently, we showed that Ca^2+^ binding of the MICU1–MICU2 heterodimer is not an independent process but a cooperative one. This result is consistent with the prior biochemical studies which confirmed the cooperative Ca^2+^ binding of the MICU1–MICU2 heterodimer using tryptophan fluorescence with a Hill coefficient of *n*_H_ = 2.1 ± 0.2 (Kamer *et al.*, 2017 ▸).

The MICU1–MICU2 heterodimer showed a lower Ca^2+^ binding affinity (*K*_d_ = 620 n*M*) compared with that of the MICU1 homodimer (*K*_d_ ≃ 300 n*M*). As shown in Fig. 3 ▸, Asp231(MICU1) located in EF-hand 1 makes a salt bridge with Arg352(MICU2) in the apo state. In addition, carbonyl oxygen of the Lys228(MICU1) backbone forms a hydrogen bond with Arg352(MICU2). Arg352(MICU2) is one of the essential residues for heterodimer formation. We expect that the interaction network mediated by Arg352(MICU2) might be reorganized in the Ca^2+^-bound structure because Asp231(MICU1) participates in Ca^2+^ coordination in the Ca^2+^-bound state [Fig. 4 ▸(*d*)]. It is reasonable to suppose that the binding interface around Asp231(MICU1)–Arg352(MICU2), which exists only in the MICU1–MICU2 heterodimer, should change when the MICU1–MICU2 heterodimer binds to Ca^2+^. The tight interaction in the apo state of MICU1–MICU2 might hinder the conformational changes required for Ca^2+^ binding, resulting in a lower Ca^2+^ binding affinity in the MICU1–MICU2 heterodimer compared with that of the MICU1 homodimer.

## 3. Discussion   

MICUs form homo- or hetero-oligomers, recognize the extra-mitochondrial [Ca^2+^] and regulate the MCU channel. Although expression levels of each MICU vary between tissues (Plovanich *et al.*, 2013 ▸; Paillard *et al.*, 2017 ▸; Patron *et al.*, 2019 ▸), MICUs in cells generally form MICU1–MICU2 heterodimers, which is consistent with prior biochemical studies showing that the MICU1–MICU2 heterodimer is more stable (*K*_d_ = 224 n*M* in the apo state) compared with homodimers of MICU1 and MICU2 (Wagner *et al.*, 2015 ▸; Patron *et al.*, 2014 ▸; Matesanz-Isabel *et al.*, 2016 ▸; Mallilankaraman *et al.*, 2012*b* ▸; Kamer & Mootha, 2014 ▸; Csordás *et al.*, 2013 ▸; Ahuja & Muallem, 2014 ▸; Wu *et al.*, 2019 ▸; Kamer *et al.*, 2017 ▸). In particular, it has been reported that MICU2 functions as a gatekeeper of the MCU for suppressing the cellular damage caused by excessive mitochondrial Ca^2+^ influx (Kamer & Mootha, 2014 ▸; Patron *et al.*, 2014 ▸). Based on these findings, the importance of MICU heterodimers has been highlighted. However, the Ca^2+^ gatekeeping mechanism of MICUs has not yet been reported. Thus, structural studies of the MICU1–MICU2 heterodimer are important for understanding the differential function of the heterodimer and the MICU1 homodimer.

The F–F interface of the MICU1–MICU2 heterodimer was similar to the other F–F interfaces of homodimers in MICUs that are biologically relevant. The primary interactions of MICU1 and MICU2 in the heterodimer were two salt bridges, Arg221(MICU1)–Asp330(MICU2) and Asp231(MICU1)–Arg352(MICU2). The Arg221(MICU1)–Asp330(MICU2) salt bridge was structurally conserved with the Arg221–Asp376 salt bridge in the MICU1 homodimer. The Arg221–Asp376 salt bridge plays an essential role in MICU dimerization and MCU activation (Wang *et al.*, 2014 ▸). Similarly, Arg221(MICU1)–Asp330(MICU2), in the heterodimer, would also be important for the heterodimerization and function, as consistent with previous results (Li *et al.*, 2016 ▸). In the case of the other salt bridge, Asp231(MICU1)–Arg352(MICU2), Wu *et al.* already reported that this salt bridge is essential for the heterodimerization of MICU1 and MICU2 in the apo state (Wu *et al.*, 2019 ▸). Thus, we assumed that the F–F interface of the MICU1–MICU2 heterodimer is biologically relevant.

The MICU1–MICU2 heterodimer had an asymmetric interface that induced the topological diversity of the heterodimer in an ASU. Compared with interface 2, interface 1 had a wider surface area, stronger binding energy and less structural fluctuation, as seen from ensemble refinement [Figs. S2 and 2 ▸(*d*)]. Interestingly, the rigid interface 1 included an Asp231(MICU1)–Arg352(MICU2) salt bridge. Asp231 is a highly conserved residue for Ca^2+^ coordination in MICU1 EF-hand 1. Thus, MICU1 EF-hand 1 can bind calcium when the salt bridge dissociates. The tight interaction in the apo state of MICU1–MICU2 might hinder the conformational changes required for the Ca^2+^ binding, resulting in a lower Ca^2+^ binding affinity in the MICU1–MICU2 heterodimer as compared with that of the MICU1 homodimer. Consequently, the MCU Ca^2+^ gatekeeping threshold was shifted to the higher concentration of Ca^2+^ by the MICU1–MICU2 heterodimer, as compared with that of the MICU1 homodimer.

In this study, we determined the MICU1–MICU2 heterodimer structure without the C-terminal helix and proposed the mechanism of Ca^2+^ threshold shift. Nevertheless, the C-terminal helix is important for understanding the MCU regulation mechanism. MICU proteins are known to interact with other MICUs or the MCU through the C-terminal helix (Kamer & Mootha, 2014 ▸; Petrungaro *et al.*, 2015 ▸; Patron *et al.*, 2014 ▸). In particular, the C-terminal helix of all human MICU proteins has a cysteine residue for di­sulfide bond formation, and Mia40 has been reported to aid di­sulfide bond formation. In addition to the C-terminal helix, MICU1 is known to be involved in the MCU complex through the interaction between the EMRE C-terminal poly acidic region and its N-terminal poly basic region (Sancak *et al.*, 2013 ▸; Tsai *et al.*, 2016 ▸). The direct interaction of MICU1 and the MCU, through basic residues of MICU1 and the aspartate ring of the MCU, was also reported (Phillips *et al.*, 2019 ▸; Paillard *et al.*, 2018 ▸). However, the underlying mechanism of how MICU interacts with the MCU–EMRE and regulates the MCU Ca^2+^ gatekeeping threshold remains unclear. In addition, although a B–B interaction between MICU2 was observed in our structure, it is unclear how the B–B interaction affects Ca^2+^ uptake by the MCU (Xing *et al.*, 2019 ▸). Thus, to understand the structural and functional role of MICUs, the structure of the MCU–EMRE–MICU complex needs to be determined.

## 4. Materials and methods   

### 4.1. Expression and purification of MICU constructs   

The human MICU1 (NM_01195518.1) (residues 85–476) and human MICU1 ΔC-terminal helix (ΔC helix) (residues 97–444) constructs were cloned into a modified pET28a vector (Novagen) with a hexahistidine (6× His) tag and a TEV protease cleavage site at the N terminal. The vectors were transformed into *Escherichia coli* (*E. coli*) BL21 (DE3) and overexpressed by induction with 0.2 m*M* iso­propyl-1-thio­galacto­pyran­oside (IPTG) at 20°C for 16 h.

The human MICU2 (NM_152726.3) (residues 84–434), human MICU2 ΔC helix (residues 84–401) and human MICU2 EF-hand 1 and 4 double mutant (residues 84–434; D185A, E196K, D375A and E386K) constructs were cloned into a pCold II vector (TaKaRa) with a 6× His tag at the N terminals. The vectors were transformed into *E. coli* BL21(DE3) (MICU2 and MICU2 EF-hand mutant) or *E. coli* B834(DE3) (MICU2 ΔC helix), and overexpressed by induction with 0.2 m*M* IPTG at 37°C for 4 h.

The cells were harvested by centrifugation and were resuspended in buffer A (50 m*M* Tris–HCl pH 7.2, 500 m*M* NaCl, 5 m*M* imidazole, 0.5 m*M* EGTA, 5 m*M* β-mercapto­ethanol and 0.3% Triton X-100). The cell suspension was disrupted by sonication and the cell lysate was centrifuged at 15 814*g* for 1 h. The supernatants containing MICU proteins were loaded onto a gravity-flow column (Bio-Rad) packed with Ni-IDA agarose resin (Elpis) pre-equilibrated with buffer A, and was subsequently washed with buffer B (20 m*M* Tris–HCl pH 7.2, 500 m*M* NaCl, 50 m*M* imidazole and 5 m*M* β-mercapto­ethanol) to remove the non-specific binding proteins. The proteins were eluted using buffer C (20 m*M* Tris–HCl pH 7.2, 300 m*M* NaCl, 500 m*M* imidazole and 5 m*M* β-mercapto­ethanol), and the TEV sites of MICU1 and MICU1 ΔC helix were cleaved by TEV protease at 4°C for 16 h (the MICU2 and MICU2 ΔC helix proteins were not cleaved). The proteins were further purified through SEC on a HiLoad 16/60 Superdex 200 prep (Pharmacia) column with SEC buffer (20 m*M* MES–NaOH pH 6.8, 300 m*M* NaCl, 5 m*M* EGTA and 5 m*M* di­thio­threitol). The collected fractions containing MICU1, MICU1 ΔC helix, MICU2, MICU2 EF-hand mutant and MICU2 ΔC helix were concentrated using an Amicon Ultra-15 30 K (Millipore) up to 3.0 mg ml^−1^. The final MICU1 or MICU2 proteins were stored at −80°C.

### 4.2. Analytical SEC of the wild-type and mutant MICU1–MICU2 heterodimer   

To evaluate the cooperative characteristics of MICU1–MICU2 heterodimer formation, we performed analytical SEC with the WT MICU1 (residues 85–476) and the WT or EF-hand double mutant (D185A, E196K, D375A and E386K) MICU2 (residues 84–434). Separately purified MICU1 and MICU2 were mixed at a molar ratio of 1:1, and incubated for at least 30 min after the addition of 5 m*M* CaCl_2_ or EGTA. Each sample of the MICU1–MICU2 heterodimer was injected into a Superdex 200 10/300GL column (GE healthcare) pre-equilibrated with a buffer (20 m*M* MES–NaOH pH 6.8, 300 m*M* NaCl, 5 m*M* di­thio­threitol, and 5 m*M* CaCl_2_ or EGTA). The molecular weight of the eluates was calculated from a standard curve generated using a standard protein kit (GE Healthcare) (Fig. S7). The eluted fractions from the same elution volume were analyzed using SDS–PAGE and Coomassie blue staining.

### 4.3. Crystallization of the human MICU1 ΔC and MICU2 ΔC complex   

For the heterodimerization of MICU1 and MICU2, proteins were mixed at a 1:1 molar ratio. The mixed proteins were initially screened for crystallization using the sitting-drop vapor-diffusion method in a 96-well INTELLI-PLATE (Art Robbins Ins.). The MICU1–MICU2 heterodimer formed rod-shaped crystals after three days in a reservoir solution containing Index I and II Screen (Hampton Research), 25%(*w*/*v*) PEG 3350, and 0.2 *M* ammonium citrate tribasic (pH 7.5). Additional crystallization trials were performed using the sitting-drop vapor-diffusion method. Finally, the optimized heterodimer crystals were grown at 20°C in 2 µl drops containing equal volumes of protein and reservoir solution with 20%(*w*/*v*) PEG 3350 and 0.2 *M* ammonium citrate tribasic (pH 7.5). For data collection, the heterodimer crystals were cryoprotected by transferring them into a cryoprotectant containing 20%(*v*/*v*) ethyl­ene glycol and flash cooling in liquid nitro­gen.

### 4.4. Data collection, structure determination and refinement   

Diffraction data of the MICU1–MICU2 heterodimer crystals were collected at 100 K using synchrotron X-ray sources on beamline 7A at the Pohang Accelerator Laboratory (PAL) (Pohang, South Korea). We collected the diffraction data for the heterodimer at a resolution of 3.1 Å using multiple wavelengths (0.9792 and 0.9794 Å). The diffraction data were processed and scaled using *iMOSFLM* (Battye *et al.*, 2011 ▸), followed by quick scaling with *POINTLESS* and *AIMLESS* in the *CCP*4 package (Winn *et al.*, 2011 ▸; Evans, 2011 ▸, 2006 ▸). Heavy-atom substructure was determined with *SHELXD* in the *CCP*4*i*2 package (Sheldrick, 2010 ▸; Potterton *et al.*, 2018 ▸). Phasing and density modification were performed with *SHELXE* in the *CCP*4*i*2 package (Sheldrick, 2010 ▸; Potterton *et al.*, 2018 ▸). Model building and phase refinement were performed with *Parrot*, *REFMAC5* and *Buccaneer* in the *CCP*4*i*2 package (Skubák & Pannu, 2013 ▸; Potterton *et al.*, 2018 ▸). In the initial model, the MICU1–MICU2 heterodimer structure was partially built. Using this initial MICU1–MICU2 heterodimer structure, we performed additional model building using the *Coot* program (Emsley & Cowtan, 2004 ▸). After the additional model building, iterative refinement was performed with *phenix.refine*, *REFMAC5* and *Coot* (Afonine *et al.*, 2013 ▸, 2009 ▸, 2012 ▸; Headd *et al.*, 2012 ▸; Liebschner *et al.*, 2019 ▸; Murshudov *et al.*, 2011 ▸; Emsley & Cowtan, 2004 ▸). The details of the data-collection and refinement statistics are provided in Table 1 ▸.

### 4.5. Structural analysis   

All the structural figures were generated using *PyMOL* version 1.5.0.4 (Schrödinger LLC) and *Coot* (Emsley & Cowtan, 2004 ▸). Multiple sequence alignment was performed using *ESPript* 3.0 (*ESPript*; http://espript.ibcp.fr; Robert & Gouet, 2014 ▸). PDBePISA was used for interface analysis (Krissinel & Henrick, 2007 ▸) and *PRODIGY* was used for the prediction of binding energies (Vangone & Bonvin, 2015 ▸; Xue *et al.*, 2016 ▸).

### 4.6. Ensemble refinement   

Ensemble refinement for MICU1 and MICU2, in the MICU1–MICU2 heterodimer, was performed using structures and structure factors by *phenix.ensemble_refinement* (Burnley *et al.*, 2012 ▸). Default parameters were used in the *phenix.ensemble_refinement*, including *pTLS* = 0.8 and *T*_bath_ = 5 K, and solvent was updated every 25 cycles. The simulations have an equilibration phase (10τ*x*) in which the temperature, X-ray weight and averaged structure factors stabilize, followed by an acquisition phase (10τ*x*). The output structures of ensemble refinement were visualized using *PyMOL* version 1.5.0.4 (Schrödinger LLC) with the script *ens_tool.py*.

## Supplementary Material

Supporting figures. DOI: 10.1107/S2052252520001840/tj5030sup1.pdf

PDB reference: crystal structure of the mitochondrial calcium uptake 1 and 2 heterodimer (MICU1–MICU2 heterodimer) in an apo state, 6le5

## Figures and Tables

**Table 1:** Data-collection and refinement statistics Values in parentheses are for the highest-resolution shell.

Protein	Apo MICU1–MICU2 heterodimer
PDB ID	6le5
	
Data collection	
Space group	*P*2_1_
X-ray source†	PAL-7A
Detector	ADSC Q270
Wavelength (Å)	0.9792
Unit-cell dimensions *a*, *b*, *c* (Å)	62.97, 173.72, 148.00
Resolution range (Å)	86.86–3.10 (3.18–3.10)
*R*_merge_ ‡	0.256 (1.898)
CC_1/2_ (%)§	0.992 (0.258)
〈*I*/σ(*I*)〉	6.2 (1.1)
Completeness (%)	100.0 (100.0)
Redundancy	7.2 (7.3)
	
Refinement	
Resolution range (Å)	50.01–3.10
No. of reflections	54709
*R*_work_ (%)/*R*_free_ (%)¶	29.3/33.2
No. of atoms/residues of protein	17392/2217
*B* factors (Å^2^) of protein	75.7
	
Model statistics	
RMSD bond lengths (Å)	0.01
RMSD bond angles (°)	1.34
Ramachandran plot (%)	
Favored/allowed/disallowed	93.8/6.2/0.0

† Beamline 7A at the PAL in South Korea.

‡ *R*_merge_ = ∑*_h_*∑*_i_* |*I*(*h*)*_i_*−〈*I*(*h*)〉|/[∑*_h_*∑*_i_* *I*(*h*)*_i_*], where *I*(*h*) is the intensity of reflection of *h*, ∑*_h_* is the sum over all reflections and ∑*_i_* is the sum over *i* measurements of reflection *h*.

§ CC_1/2_ was calculated from *MOSFLM* (Battye *et al.*, 2011 ▸).

¶ *R*_work_ = ∑*_hkl_* ||*F*_o_| − |*F*_c_||/(∑*_hkl_* |*F*_o_|); 5% of the reflections were excluded for the *R*_free_ calculation.
