# Supplementary material for: Structure of the MICU1–MICU2 heterodimer provides insights into the gatekeeping threshold shift
Source: IUCrJ. 2020 Feb 27;7(Pt 2):355–65. doi: 10.1107/S2052252520001840 (PMC7055370; doi:10.1107/S2052252520001840)
Supplement: Supplementary file 1 [file m-07-00355-sup1.pdf]

IUCrJ

Volume 7 (2020)

Supporting information for article:

Structure of the MICU1–MICU2 heterodimer provides insights into the gatekeeping threshold shift

Jongseo Park, Youngjin Lee, Taein Park, Jung Youn Kang, Sang A Mun, Minwoo Jin, Jihyeong Yang and Soo Hyun Eom

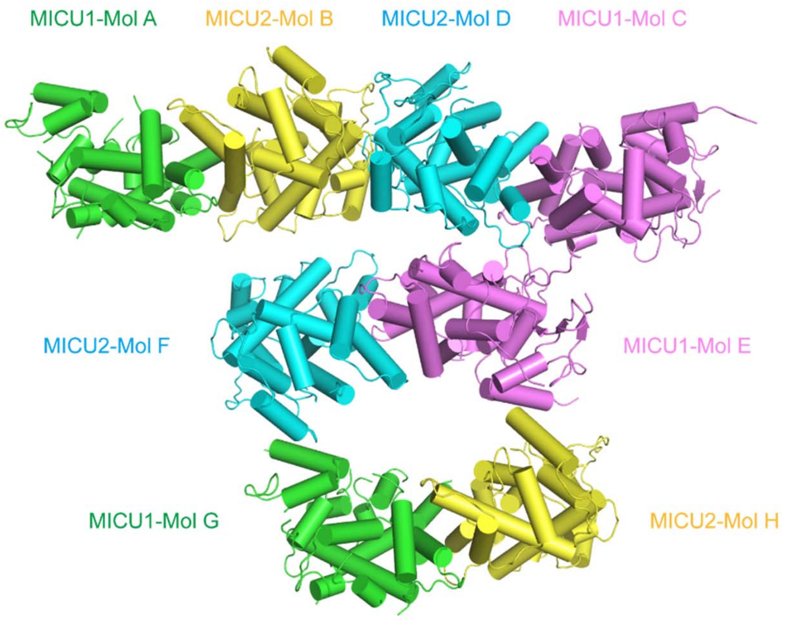

Figure S1 Four heterodimers consisting of an asymmetric unit. Overall view of the MICU1-MICU2 heterodimer structure occupying the ASU. There are four heterodimers which interact with each other through back-to-back interaction of MICU2. MICU1 is colored in green and violet. MICU2 is colored in yellow and cyan.

Interface area	Face-to-Face (F-F) dimer			Back-to-Back (B-B) dimer	
	MICU1-2	MICU1 (4NSC)	MICU2 (6AGH)	MICU2	MICU2 (6AGH)
Interface 1 (Å)	654	532	361	860	1260
Interface 2 (Å)	346	596	406		

(a)

Predicted Binding Energy (F-F dimer)	MICU1-2	MICU1 (4NSC)	MICU2 (6AGH)
Interface 1 (kcal/mol)	-7.6	-6.9	-5.5
Interface 2 (kcal/mol)	-5.2	-7.0	-6.0

(b)

Figure S2 Interface area and predicted binding energies of the MICU1-MICU2 heterodimer and other homodimers in MICUs. **(a)** The interface area of the MICU1-MICU2 heterodimer and F-F or B-B MICU homodimers calculated using the PDBePISA web server (PDB ID: 4NSC and 6AGH). MICU1-MICU2 heterodimer (Mol CD) and MICU2 homodimer (Mol BD) in the ASU are abbreviated to MICU1-2 and MICU2, respectively. Only the total interface area is provided in the B-B dimer. **(b)** Predicted binding energies (ΔG, kcal/mol) calculated using the PRODIGY web server. Interface area and predicted binding energies of MICU1-MICU2 heterodimer (Mol AB, CD), MICU1 homodimer (Chain AB, CE, DF), and B-B MICU2 homodimers in the ASU (Mol BD, Mol FH in crystal lattice) were calculated for each average.

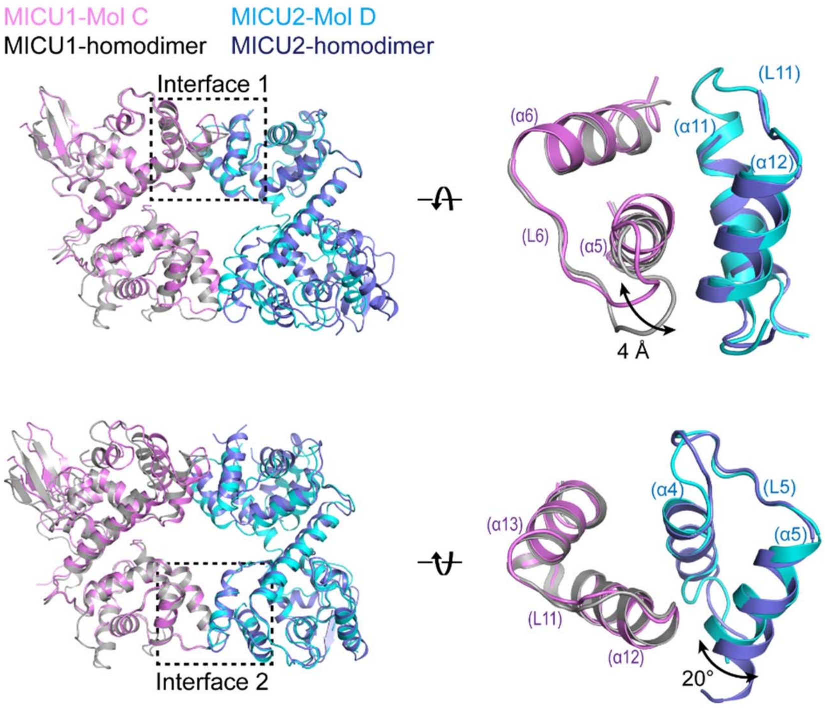

Figure S3 Conformational change of interface 1 and 2 compared to the apo state of MICU1 and MICU2. Cartoon representation of interface 1 and 2 of superimposed MICU1-MICU2 heterodimer and homodimers in MICU1 and MICU2 based on each interface. The conformational change of interloop (L6) of MICU1 EF-hand 1 and helix α5 of MICU2 EF-hand 3 is indicated with black arrow.

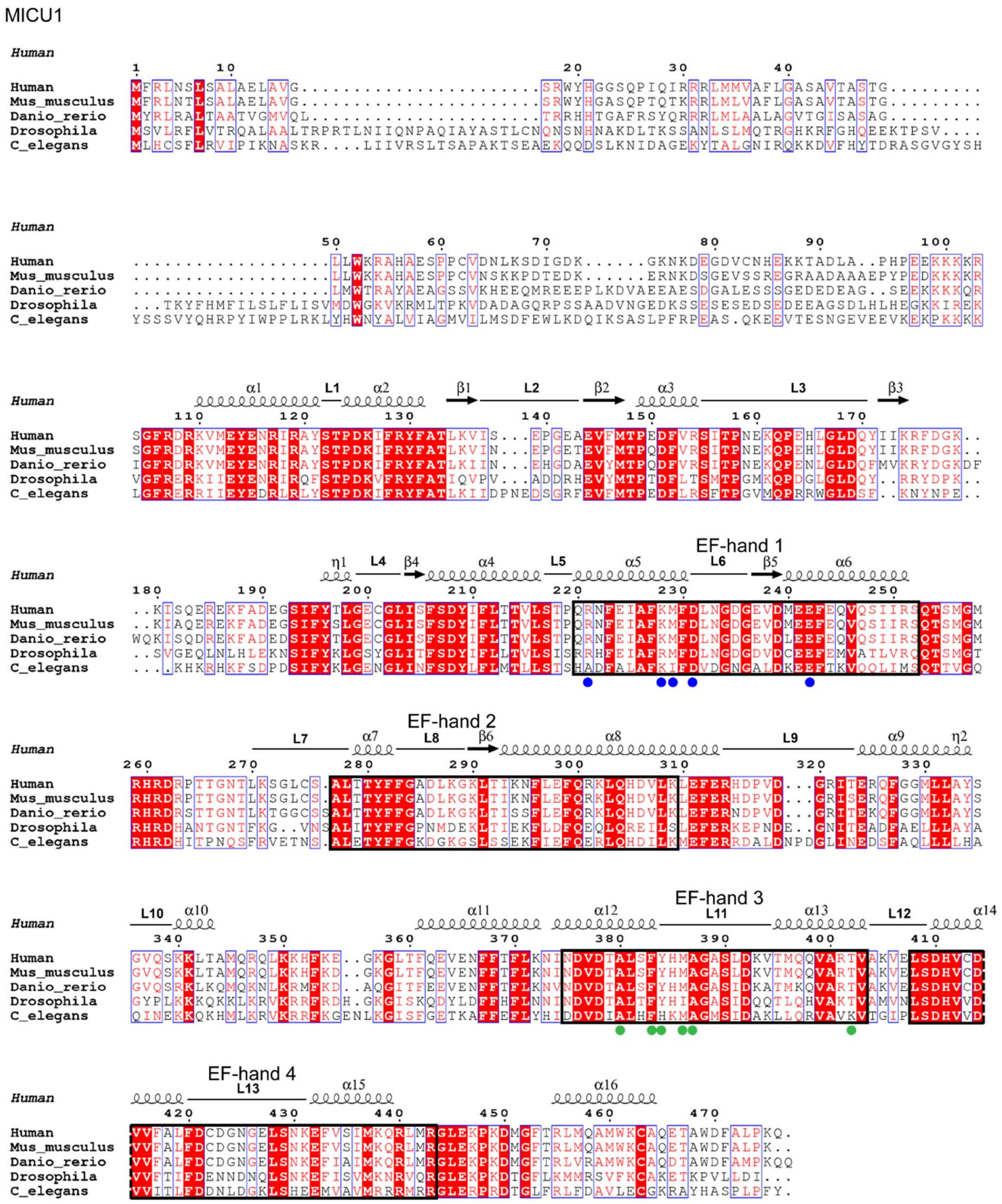

Figure S4 Multiple sequence alignment of MICU1. The amino acid sequence alignment of MICU1 sequences from various species. Key residues of interface 1 and 2 are marked by blue and green dots, respectively. All of the EF-hands are marked by black solid lined box.

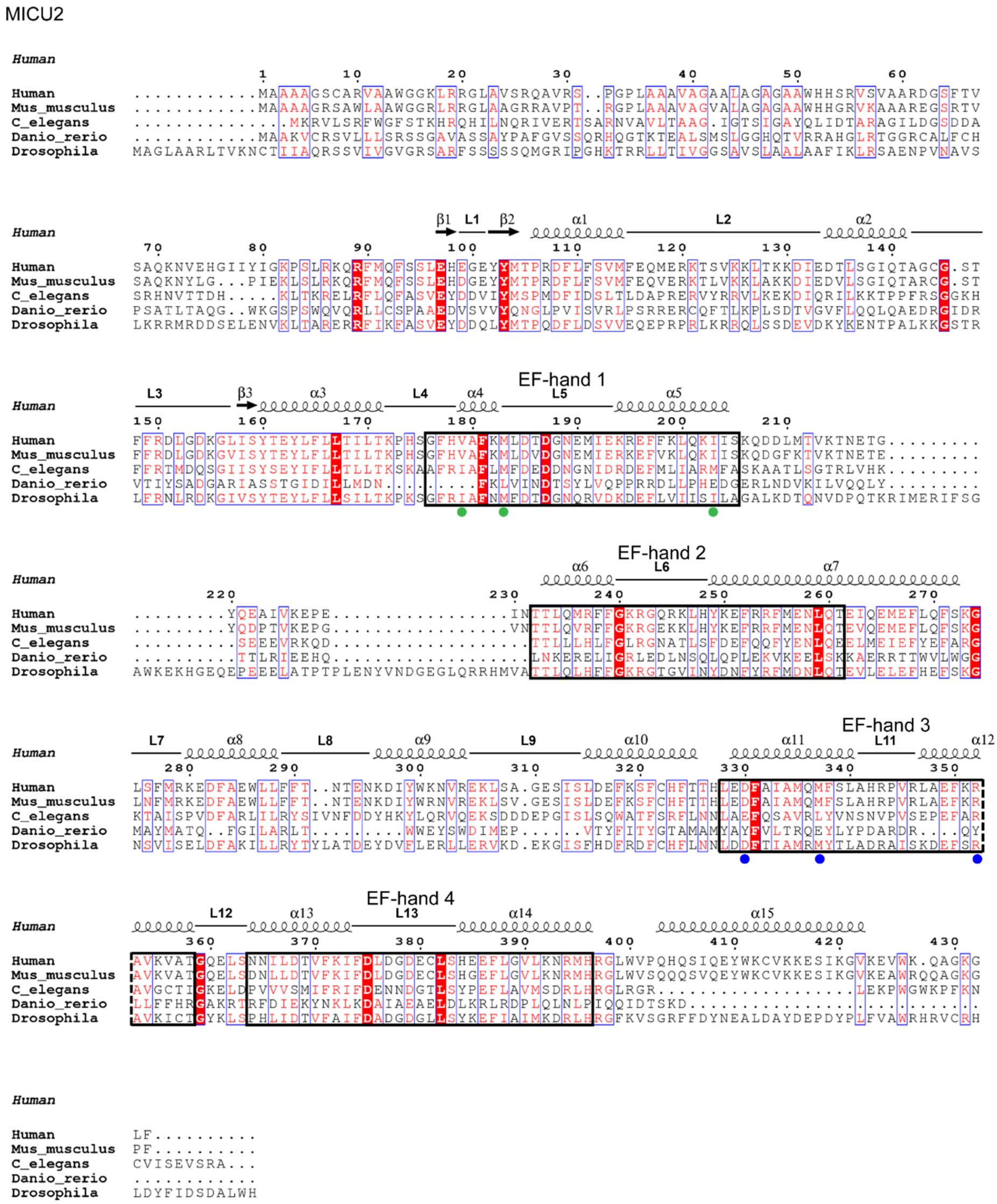

Figure S5 Multiple sequence alignment of MICU2. The amino acid sequence alignment of MICU2 sequences from various species. Key residues of interface 1 and 2 are marked by blue and green dots, respectively. All of the EF-hands are marked by black solid lined box.

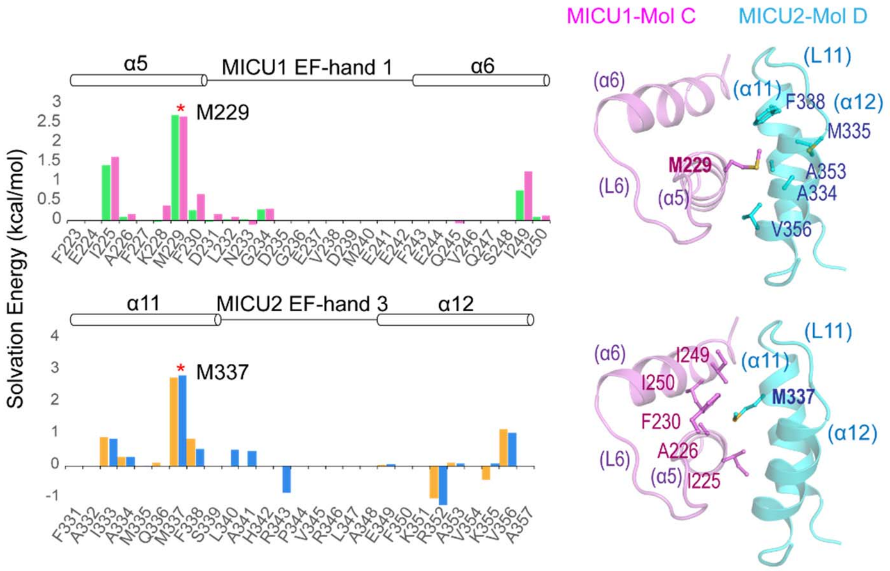

Figure S6 Solvation energies and cartoon representations of MICU1 and MICU2 in interface 1. Plots of solvation energy (kcal/mol) and cartoon representations for EF-hand 1 and EF hand 3 of MICU1 or MICU2 constituting interface 1. Cartoon representations show the several residues of interface 1 participating in hydrophobic interactions with two methionine knobs of MICU1-MICU2 heterodimer, Mol CD. Asterisks show the residues that have the highest solvation energy value in each EF-hand, M229(MICU1) and M337(MICU2).

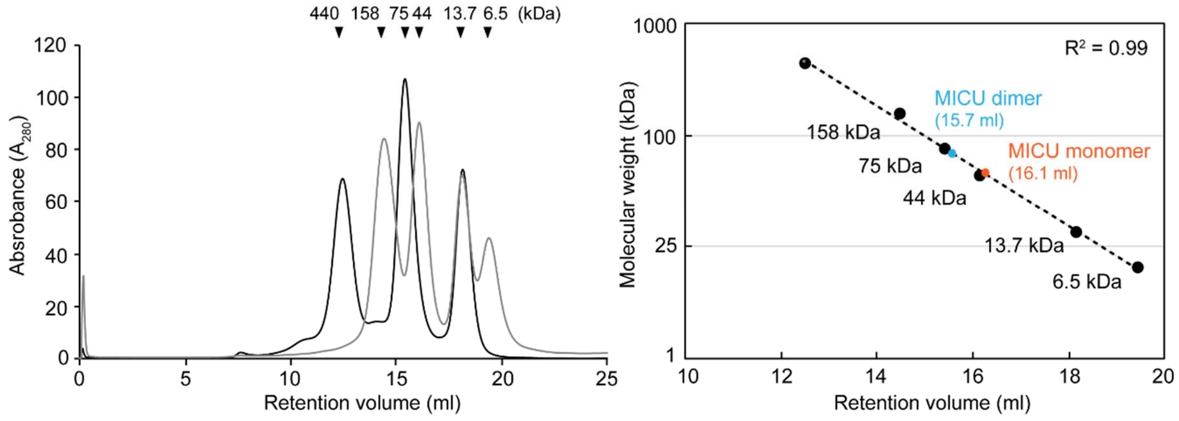

Figure S7 Calibration chromatogram and standard curve of size-exclusion chromatography (SEC). **(a)** Size-exclusion chromatogram of molecular weight (m.w.) standards containing ferritin (440 kDa), aldolase (158 kDa), Conalbumin (75 kDa), Ovalbumin (43 kDa), ribonuclease A (13.8 kDa), and aprotinin (6.5 kDa). **(b)** A standard curve based on the chromatogram. Cyan and orange dots indicate the eluted position of MICU dimer (89.7 kDa) and MICU monomer (44.9 kDa), respectively.
